# The generalized Shockley-Queisser limit for nanostructured solar cells

**DOI:** 10.1038/srep13536

**Published:** 2015-09-02

**Authors:** Yunlu Xu, Tao Gong, Jeremy N. Munday

**Affiliations:** 1 Department of Electrical and Computer Engineering, University of Maryland, College Park, MD 20740, USA; 2 Institute for Research in Electronics and Applied Physics, University of Maryland, College Park, MD 20740, USA

## Abstract

The Shockley-Queisser limit describes the maximum solar energy conversion efficiency achievable for a particular material and is the standard by which new photovoltaic technologies are compared. This limit is based on the principle of detailed balance, which equates the photon flux into a device to the particle flux (photons or electrons) out of that device. Nanostructured solar cells represent a novel class of photovoltaic devices, and questions have been raised about whether or not they can exceed the Shockley-Queisser limit. Here we show that single-junction nanostructured solar cells have a theoretical maximum efficiency of ∼42% under AM 1.5 solar illumination. While this exceeds the efficiency of a non-concentrating planar device, it does not exceed the Shockley-Queisser limit for a planar device with optical concentration. We consider the effect of diffuse illumination and find that with optical concentration from the nanostructures of only × 1,000, an efficiency of 35.5% is achievable even with 25% diffuse illumination. We conclude that nanostructured solar cells offer an important route towards higher efficiency photovoltaic devices through a built-in optical concentration.

In 1961, Shockley and Queisser developed a theoretical framework for determining the limiting efficiency of a single junction solar cell based on the principle of detailed balance equating the incoming and outgoing fluxes of photons for a device at open-circuit conditions^1^. This model incorporates various light management and trapping techniques including photon recycling, optical concentration, and emission angle restriction^1^,^2^,^3^. It was recently suggested that a nanowire solar cell could exceed the Shockley-Queisser (SQ) limit based on its geometry^4^; however, without exploiting 3rd generation photovoltaic (PV) concepts that break the assumptions of Shockley and Queisser (e.g. multi-exciton generation, hot carrier collection, etc)^5^,^6^,^7^, even nanowire solar cells should be bounded by the SQ limit. Here we show that for *any* nanostructured solar cell (e.g. composed from wires, cones, pyramids, etc.), the limiting efficiency is identical to that of a planar solar cell with concentrating optics and that the improvement results strictly from an increase in the open-circuit voltage. This formalism leads to a maximum efficiency of ∼42% for a nanostructured semiconductor with a bandgap energy of ∼1.43 eV (e.g. GaAs) under AM 1.5G illumination^8^.

The SQ limit is reached by applying the principle of detailed balance to the particle flux into and out of the semiconductor^1^. For every above bandgap photon that is absorbed by the semiconductor, one electron-hole pair is generated. The maximum possible efficiency is achieved when non-radiative recombination is absent, and all generated carriers are either collected as current in the leads or recombine, emitting a single photon per electron-hole pair. The total generated current is:


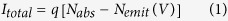


where *q* is the charge of an electron, and *N*_*abs*_ and *N*_*emit*_ are the numbers of photons per unit time that are absorbed or emitted by the photovoltaic device, respectively. These rates can be calculated as^2^:


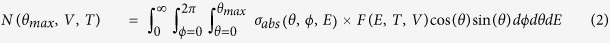


where *σ*_*abs*_(*θ*, *ϕ*, *E*) is the absorption cross-section, *F*(*E*, *T*, *V*) is the spectral photon flux, and *θ*_*max*_ is the maximum angle for absorption (for *N*_*abs*_) or emission (for *N*_*emit*_). For a bulk planar cell, the absorption cross-section is given by *σ*_*abs*_(*θ*, *ϕ*, *E*) = *A*_*cell*_ × *a*(*θ*, *ϕ*, *E*), where *A*_*cell*_ is the top illuminated surface area of the cell and *a*(*θ*, *ϕ*, *E*) is the angle dependent probability of photon absorption for incident photons of energy *E*. In the simplest case, *a*(*θ*, *ϕ*, *E*) is a step-function going from 0 (for *E* < *E*_*g*_) to 1 (for *E* ≥ *E*_*g*_). The spectral photon flux can be obtained from the generalized Planck blackbody law^9^:


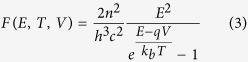


where *h* is Planck’s constant, *k*_*b*_ is Boltzmann’s constant, *c* is the speed of light, *n* is the refractive index of the surroundings, which is usually taken to be vacuum (*n* = 1), and *qV* characterizes the quasi-Fermi level splitting when describing emission from the cell. The incoming flux from the sun can be obtained from experimental data (e.g. AM 1.5 solar spectrum) or from the blackbody expression above with *V* = 0 and where *θ*_*max*_ = *θ*_*s*_ = 0.267° is the acceptance half-angle for incident light from the sun at temperature *T* = *T*_*s*_ = 5760 *K*. The outgoing flux from the cell is given by Eq. (2) for a cell temperature *T*_*c*_ = 300 *K*, operating voltage *V*, and emission half-angle *θ*_*max*_ = *θ*_*c*_ = 90°. At open-circuit conditions, there is no current extracted, and the current balance equation becomes


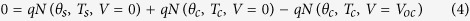


where the middle term corresponds to absorption due to emission from the ambient surroundings, also at *T* = 300 *K*; however, this term is much smaller than the flux from the sun. Thus, the light generated current is given by *I*_*L*_ = *qN* (*θ*_*s*_, *T*_*s*_, *V* = 0) and the dark current, in the radiative limit, is given by 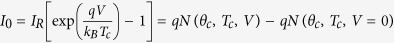, where *I*_*R*_ is the reverse saturation current. Solving Eq. (4) for the voltage yields the common expression for the open-circuit voltage^1^,^8^:


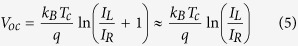


which is valid for both bulk planar solar cells and nanostructured solar cells with the appropriate absorption cross-sections as described in the next section.

## Results

### Nanostructured solar cells with built-in optical concentration

To achieve the maximum efficiency, we need to increase the light generated current compared to its bulk form or reduce the reverse saturation current to increase *V*_*oc*_. For any absorbing structure, Eqs (2, 3, 4, 5) can be used to determine the resulting *V*_*oc*_ numerically; however, for the limiting case, we will consider a simple analytical expression. For maximum *V*_*oc*_, we want the absorption cross-section to be maximized for angles near normal incidence up to an angle *θ*_*m*_ (where *θ*_*s*_ ≤ *θ*_*m*_) and minimized for all other angles *θ*_*m*_ ≤ *θ* ≤ *θ*_*c*_, where *θ*_*m*_ is some angle defined by the structure. We can define this piece-wise function for the absorption cross-section as: *σ*_*abs*_(*θ* : 0 →*θ*_*m*_) = *σ*_*max*_ and *σ*_*abs*_(*θ* : *θ*_*m*_ →*θ*_*c*_)* = σ*_*min*_, which allows us to perform the solid angle integration to determine the light and dark currents:


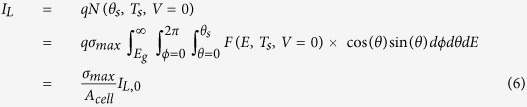


where *σ*_*abs*_ = 0 for *E* < *E*_*g*_, *I*_*L*,0_ is the light generated current for an ideal bulk cell of area *A*_*cell*_, and


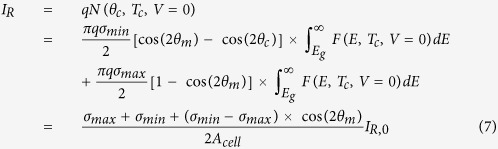


where *I*_*R*,0_ is the reverse saturation current for a bulk cell. Substituting these expressions into Eq. (5), we have


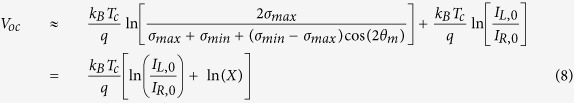


where


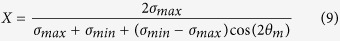


Thus, the open-circuit voltage for a nanostructured device takes on the same form as the open-circuit voltage for a macroscopic concentrating system, where *X* is the concentration factor^8^. For maximum concentration, we consider the limit as *θ*_*m *_→*θ*_*s*_ and σ_*min*_  → 0, yielding


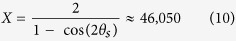


which is the same as the maximum concentration factor that is obtained for a macroscale concentrator and results in a maximum solar energy conversion efficiency of ∼42%. For practical devices it is reasonable to assume a minimum value of *σ*_*min*_ corresponding to the geometric cross-section of the device, *σ*_*min*_ →*σ*_*geo*_. For this case, and with cos(2*θ*_*m*_) = cos(2*θ*_*s*_) ≈ 1, we get *X* = *σ*_*max*_/*σ*_*geo*_, and the open-circuit voltage reduces to:


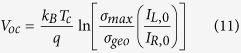


**Figure 1:**
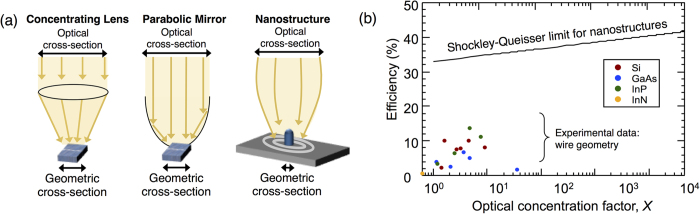
The Shockley-Queisser limit for nanostructures. (**a**) Schematic of the optical concentration implemented by a concentrating lens, parabolic mirror, and using a nanostructure itself (self-concentration). (**b**) The efficiencies of cells with optical concentration. The solid line is the theoretical limit of nanostructured PV devices based on detailed balance, whereas individual dots represents experimental data reported in the literature^4^,^10^,^11^,^12^,^13^,^14^,^15^,^16^,^17^,^18^,^19^,^20^,^21^,^22^,^23^,^24^.

Finally, the power conversion efficiency is given by *η* = *I*_*L*_*V*_*oc*_*FF*/*P*_*in*_, where *FF* is the fill-factor, which can be obtained from the *I*−*V* characteristic defined by Eq. 1, and *P*_*in*_ is the incident power from the sun. We note that the area used to calculate *P*_*in*_ is determined by the illumination area and not the geometric cross-section, which would lead to undercounting the number of incident photons. In general, optical concentration can be achieved using lenses, mirrors, or unique optical nanostructures (see Fig. 1(a)). A nanostructured solar cell can result in optical concentration that is similar to the concentration obtained using lens or parabolic mirrors but relies on the wave nature of light. Fig. 1 (b) shows the power conversion efficiency of recently reported vertically aligned nanowire-based PV cells^4^,^10^,^11^,^12^,^13^,^14^,^15^,^16^,^17^,^18^,^19^,^20^,^21^,^22^,^23^,^24^. The optical and geometrical cross-sections are extracted from the current density data and from the geometrical information provided within the references. The vast majority of the experiments are focused on Si, GaAs and InP radial or axial junction nanowire arrays fabricated with various techniques, such as MBE, MOVCD, reactive-ion etching, etc. Generally, 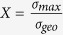 is found to fall in the range of 1–10 for these structures; however, the actual concentration factor is likely significantly smaller if *σ*_*min*_ > *σ*_*geo*_. Additionally, the reduced efficiency in these nanowire structures compared to the theoretical limit is due to significant surface recombination and device and material constraints that could be improved with further experimental development.

### The effect of entropic losses on *V*_*oc*_

Next we consider an alternative, but equivalent, approach to understanding the maximum efficiency of a nanostructured PV device by considering the energetic and entropic loss mechanisms^25^,^26^,^27^. The generalized Planck equation can be used to determine the open-circuit voltage of a solar cell operating at the maximum efficiency limit^25^,^28^,^29^:


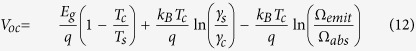


where *γ*_*s*_ and *γ*_*c*_ are blackbody radiation flux terms that depend on *E*_*g*_, *T*_*s*_, and *T*_*c*_. The first term represents a voltage drop related to the conversion of thermal energy into work (sometimes called the Carnot factor). The second term occurs from the mismatch between Boltzmann distributions at *T*_*c*_ and *T*_*s*_^30^. The third term is the voltage loss due to entropy generation as a result of a mismatch between the absorption solid angle and the emission solid angle of the cell. This third term represents a voltage drop of ∼0.28 V, which can be recovered if Ω_*emit*_ = Ω_*abs*_. Modification of the directionality of absorption and emission to improve the open-circuit voltage of a solar cell is well-known^31^,^32^,^33^ and has recently been shown in experiments^34^,^35^.

**Figure 2:**
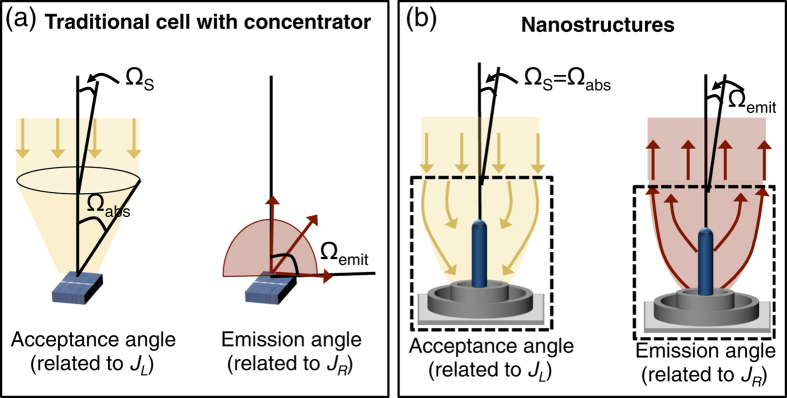
Nanostructures can reduce the mismatch between absorption and emission angles. (**a**) A traditional planar solar cell with concentrator increases Ω_*abs*_ to approach Ω_*emit*_, thus reducing the entropy generation caused by their mismatch. (**b**) Similarly, a nanostructured solar cell can reduce the difference between Ω_*abs*_ and Ω_*emit*_.

The most common way to recover the entropy loss due to the mismatch between the absorption and emission solid angles is through optical concentration (Fig. 2(a)). For a planar solar cell without optical concentration, the absorption solid angle corresponds to the sun’s angular extent, i.e. Ω_*abs*_ = 2*π*(1 − cos(*θ*_*s*_)) = 6.82 × 10^−5^ sr. However, emission from the cell occurs over Ω_*emit*_ = 4*π*. The addition of a back reflector reduces the emission solid angle to Ω_*emit*_ = 2*π*, resulting in a slight voltage improvement^2^. For more substantial voltage improvements, optical concentration is necessary. Optical concentration enables the absorption solid angle to exceed the sun’s solid angle and approach the cell’s emission solid angle (Fig. 2(a)), which could largely increase the *V*_*oc*_.

Properly designed photovoltaic nanostructures can have the same effect, reducing the entropy generation by either increasing Ω_*abs*_ or by reducing Ω_*emit*_ in an attempt to achieve Ω_*emit*_ = Ω_*abs*_ (Fig. 2(b)). From a device point-of-view, Ω_*abs*_ is related to the light generated current density, *J*_*L*_ = *I*_*L*_/*A*, and Ω_*emit*_ is related to the reverse saturation current density, *J*_*R*_ = *I*_*R*_/*A*. Because the *V*_*oc*_ depends on their ratio (see Eq. 5), increasing Ω_*abs*_ will have the same affect as decreasing Ω_*emit*_. Thus, the voltage improvement can equivalently be seen from the thermodynamics of reduced entropy generation or from the device aspects of the *p*-*n* junction.

**Figure 3:**
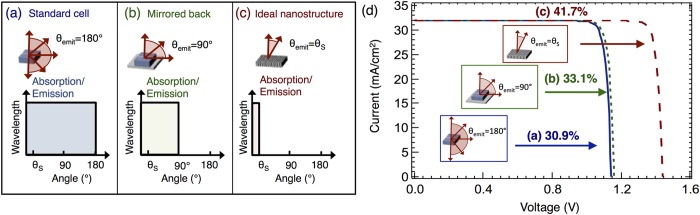
Modification of absorption and emission results in an ideal PV nanostructure achieving >40% power conversion efficiency. Emission and absorption for (**a**) slab without back reflector (i.e. light can escape through the back surface without reflection), (**b**) slab with back reflector, and (**c**) ideal nanostructured cell. The emission and absorption are represented in terms of their half-angle, *θ*. Absorption/emission over all angles (standard cell) corresponds to *θ* = 180°; however, the illumination from the sun is only over a subset of half-angles from 0 to *θ*_*s*_. Thus, the mismatch between *θ*_*s*_ and *θ*_*emit*_ results in a decreased voltage. (**d**) I-V curves corresponding to the three structures (**a**–**c**). All structures are illuminated with the AM 1.5G spectrum and show increased *V*_*oc*_ as *θ*_*emit*_→*θ*_*s*_.

According to Kirchhoff’s law, the emissivity and absorptivity of a solar cell are equal in thermal equilibrium^2^,^36^. For a standard cell without back reflector, the device can absorb the incident power from all directions and hence will emit in all directions (Fig. 3(a)). The addition of a back reflector reduces both absorption and emission from the back surface (Fig. 3(b)); however, this has no effect on the absorption of the incident solar power because no illumination is coming from the back. Thus, *I*_*L*_ is unaffected by the addition of the back reflector but *I*_*R*_ is reduced (note: technically *I*_*L*_ could be slightly increased due to an increased path length in thin or low absorption materials, resulting in a small increase in *V*_*oc*_). An ideal nanostructure would allow for absorption only over the range of angles corresponding to the incident illumination of the source, i.e. the sun (Fig. 3(c)). The current-voltage characteristics for these devices show that a back reflector yields a ∼2% increase in efficiency over the traditional planar device, and an ideal nanostructure yields a ∼11% improvement, resulting in a ∼42% efficient device.

### Effect of diffuse illumination

**Figure 4:**
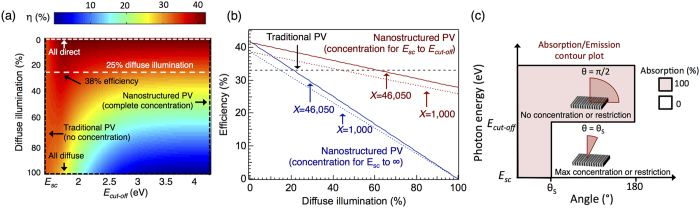
Effect of diffuse illumination. (**a**) Contour plot showing the influence of diffuse illumination on nanostructured PV as the cut-off energy for nanoscale concentration (*E*_*cut*-*off*_) is varied, assuming maximum concentration (X = 46,050). *E*_*sc*_ corresponds to the semiconductor bandgap of the device. (**b**) 3 slices of the contour plot in (a) corresponding to *E*_*cut*-*off*_ = 1.43 eV (traditional PV), *E*_*cut*-*off*_ = 1.74 eV (concentration for photons from *E*_*sc*_ to *E*_*cut*-*off*_), and *E*_*cut*-*off*_  →∞ (concentration for all incident photons); similar calculations performed for X = 1,000 are also shown. The nanostructured device with complete concentration (i.e. concentration for all energies of incident photons) outperforms traditional PV when diffuse illumination accounts for <20% of the incident light. The nanostructured device with partial concentration (corresponding to concentrating only light with energies 1.43–1.74 eV) outperforms the traditional device when the incident light is <60% diffuse. With only modest concentration (X = 1,000), the device has an efficiency of 35.5% under 25% diffuse illumination. (**c**) Absorption contour plot and schematic depicting a nanoscale device that is able to concentrate light with energies *E*_*sc*_ to *E*_*cut*-*off*_ but unable to concentrate light with energy greater than *E*_*cut*-*off*_.

While the maximum power conversion efficiency is achieved with 100% direct illumination (i.e. the incident light is completely within the solid angle defined by *θ*_*s*_), an efficiency of ∼38% can be achieved when 25% of the incident illumination is diffuse (Fig. 4), which is typical of many geographic regions. Incident illumination on earth contains both direct and diffuse components (due to scattering of the incident light). Using traditional macroscopic concentrating optics, light is concentrated for all wavelengths, and only the direct components can be used. Alternatively, nanostructures typically have a wavelength-dependent response and may only be able to concentrate light over a particular bandwidth, e.g. from the semiconductor bandgap energy (*E*_*sc*_) to some cut-off energy (*E*_*cut-off*_). This limited bandwidth for concentration is beneficial when the illumination is not 100% direct, because the diffuse components that lie outside this range can still be collected.

Figure 4 shows that efficiencies >35% can be achieved even when the illumination contains a significant fraction of diffuse light. The nanostructures depicted in Fig. 4 are able to concentrate the incident light from *E*_*sc*_ to *E*_*cut-off*_ and are unable to concentrate light with energies >*E*_*cut-off*_, which corresponds to absorption of diffuse light in that bandwidth. For *E*_*cut-off*_ = 1.74 eV, X = 1,000, and 25% diffuse illumination, the nanostructured devices reach an efficiency of 35.5%.

### Numerical simulation of nanowire PV

**Figure 5:**
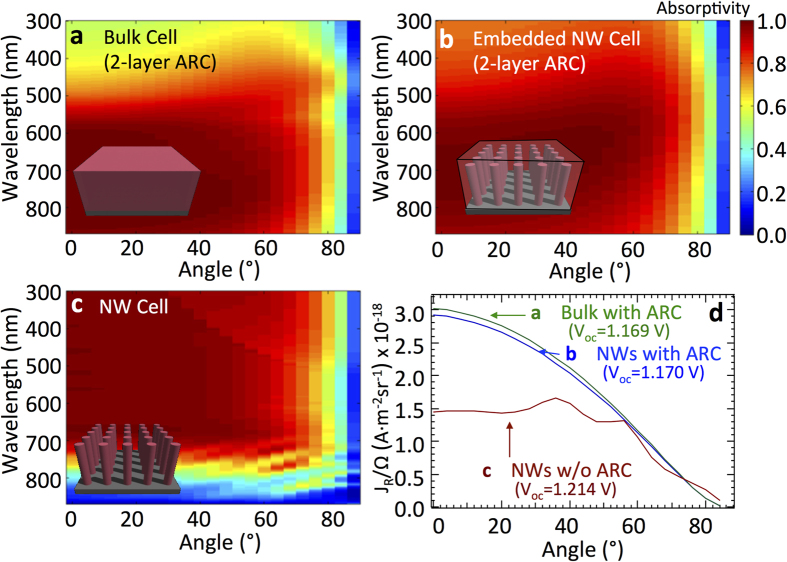
Reduced dark current in nanowire structures. Angular dependence of the absorption spectrum for (**a**) a bulk (80 *μm* thick) GaAs solar cell, (**b**) a GaAs nanowire solar cell (embedded in a dielectric) with a period of 300 nm, a radius of 75 nm, and length of 80 *μ*m, and (**c**) a GaAs nanowire solar cell with a period of 200 nm, a radius of 40 nm, and a length of 2 *μ*m. The devices in (**a**,**b**) have a double-layer ARC on top, and all cells have a perfect back reflector. The nanowire solar cells have decreased absorption (and hence emission) near the bandedge for angles >*θ*_*s*_. (**d**) The current density corresponding to the three structures (**a**–**c**) decreases, showing an improved *V*_*oc*_ for the nanowire devices.

While the above discussion is general and provides the limiting efficiency of *any* nanostructured solar cell (e.g. wires, cones, pyramids, etc.), explicate cell architectures can be studied via numerical simulation. There are no implicit assumption about the directionality of the absorption or emission; these quantities are numerically calculated directly for each structure. We have simulated a bulk (80 *μm* thick) GaAs solar cell and a nanowire solar cell with the same thickness (with periodicity of 300 *nm* and radius of 75 *nm*) using the S4 simulation package^37^ to obtain the absorption profiles. We then solved the detailed balance expression numerically^38^,^39^. A similar method was recently used to calculate the detailed balance efficiency for an InP nanowire array, and an efficiency improvement of 1.5% was reported compared to a bulk device^40^. For simplicity, we used the blackbody spectrum in the following calculations. The nanowires are embedded within a material with an index of refraction of *n* = 2.66, and both the nanowire and planar structures are coated with a double-layer antireflection coating (a 52 nm layer with *n* = 2.66 and a 98 nm layer with *n* = 1.46). The antireflection coating is designed to maximize the efficiency of the bulk GaAs cell. The integrated short circuit current density is almost identical for both cases (<1% difference); however, the emitted power density is significantly different. Because a large amount of the radiated power is near the bandgap, the lower absorption rate near the bandgap that occurs with the nanowire structure leads to a decrease in emission. This effect is demonstrated in Fig. 5(d), where the bulk cell has a higher reverse saturation current density compared to the nanowire cell with same thickness. The reverse saturation current of the nanowire cell decreases by 3.46%, and the absorption increases by 0.38%. As a result, the *V*_*oc*_ increases by 1 mV due to these combined effects in the nanowire device, and thus, the nanowire solar cell has a slightly higher efficiency than the bulk device (28.22% vs. 28.09%).

Ideally, an optical structure should be designed to minimize absorption for angles greater than *θ*_*s*_, particularly near the semiconductor bandgap, which is where the emission is peaked. To emphasize this effect, we consider a smaller radius nanowire (40 nm), which will have increased optical concentration. In order to minimize the loss in photogenerated current, the periodicity is decreased to 200 nm, and the nanowire length is set to 2 *μ*m, which is a reasonable thickness for a GaAs cell. Figure 5(c) shows this device whose absorption near the bandgap is limited so that the reverse saturation current density is one order of magnitude smaller than that of the bulk cell (Fig. 5(d)). This nanostructuring leads to the reverse saturation current decreasing from 8.751 × 10^−18^ to 9.946 × 10^−19^ A/m^2^. Although the absorption is also decreased (*J*_*L*_ decreased from 362.68 to 237.55 A/m^2^), the *V*_*oc*_ is increased from 1.169 V to 1.214 V, showing an improvement of 45 mV in *V*_*oc*_. This result suggests that nanostructures that incorporate more complexity may yield higher *V*_*oc*_’s without loss in *I*_*L*_.

## Discussion

While the overall performance of nanostructured solar cells is still bounded by the SQ limit, one must consider the built-in optical concentration when applying this theory. Recently an InP nanowire solar cell was found to have a *V*_*oc*_ in excess of the record InP planar device^21^,^41^. This improvement is likely the result of the built-in optical concentration, which leads to higher carrier densities and hence a higher *V*_*oc*_. Although the best devices to date are <14% efficient^4^,^10^,^11^,^12^,^13^,^14^,^15^,^16^,^17^,^18^,^19^,^20^,^21^,^22^,^23^,^24^, there is great potential for improvement, which could allow nanowire solar cells to exceed 40% solar power efficiency. Here we have shown that besides the possibility of improved carrier collection that has been previously reported^42^,^43^,^44^, another key advantage of nanostructured solar cells over planar ones is that the optical concentration is already built-in, yielding the possibility of higher efficiencies than planar devices.

The main limitations for exploiting these concepts in practical devices lie in minimization of non-radiative recombination and achieving appropriate optical design. Minimizing both surface and bulk non-radiative recombination is important for all PV technologies, and great strides have been achieved recently. GaAs has been shown to have an internal luminescence efficiency of >99%, leading to solar cells that operate in the radiative limit^45^,^46^, a key requirement for exploiting the concepts discussed in this manuscript. For nanostructured PV, non-radiative recombination is likely dominated by surface recombination. InP has shown excellent promise for nanostructured PV with unpassivated nanowire structures yielding surface recombination velocities as low as 170 cm/s^47^,^48^. Finally, implementation of high quality optical structures with the appropriate angular and frequency dependence may be further guided by concepts from metamaterials, metasurfaces, and transformation optics, which have previously yielded broadband angular selectivity^49^,^50^.

In conclusion, we have used the principle of detailed balance to determine the maximum efficiency for nanostructured photovoltaic devices. Because the principle of detailed balance requires knowledge of the absorption within the structure rather than the detailed geometry or arrangement, any specific nanostructure (regardless of configuration) will be bounded by this limit. The role of the geometry, period, disorder, etc. are all included by considering the absorption spectrum. The ideal nanostructured devices result in an efficiency of 42%, which is equivalent to the result of Shockley and Queisser when considering full optical concentration. This improvement comes strictly from an improvement of the open-circuit voltage, and not from an improvement in the current. We have assumed that the cell is limited by radiative emission and is under direct illumination in order to achieve the maximum efficiency limit. As with other forms of optical concentration, the efficiency is reduced if part of the incident illumination is diffuse (e.g. if 25% of the incident light is diffuse, the maximum efficiency is reduced to 38%). For future nanostructured devices to take advantage of these benefits, high quality surface passivation and reduced non-radiative recombination are needed. From an optical design point-of-view, nanostructures should be created that have limited absorption for angles and wavelengths that do not match the incident illumination. When this condition is achieved, new high efficiency nanostructured PV devices will be possible.

## Additional Information

**How to cite this article**: Xu, Y. *et al.* The generalized Shockley-Queisser limit for nanostructured solar cells. *Sci. Rep.* **5**, 13536; doi: 10.1038/srep13536 (2015).

## References

[b1] Shockley W. & Queisser H. J. Detailed Balance Limit of Efficiency of p-n Junction Solar Cells. J. Appl. Phys. 32, 510–519 (1961).

[b2] Marti A., Balenzategui J. L. & Reyna R. F. Photon recycling and Shockley’s diode equation. J. Appl. Phys. 82, 4067–4075 (1997).

[b3] Munday J. N. The effect of photonic bandgap materials on the Shockley-Queisser limit. J. Appl. Phys. 112, 064501 (2012).

[b4] Krogstrup P. *et al.* Single-nanowire solar cells beyond the Shockley-Queisser limit. Nat. Photonics 7, 306–310 (2013).

[b5] Green M. A. Third generation photovoltaics: Ultra-high conversion efficiency at low cost. Prog. Photovolt. Res. Appl. 9, 123–135; 10.1002/pip.360 (2001).

[b6] Ross R. T. & Nozik A. J. Efficiency of hot-carrier solar energy converters. J. Appl. Phys. 53, 3813–3818 (1982).

[b7] Hanna M. C. & Nozik A. J. Solar conversion efficiency of photovoltaic and photoelectrolysis cells with carrier multiplication absorbers. J. Appl. Phys. 100, 074510 (2006).

[b8] Luque A. & Hegedus S. Handbook of Photovoltaic Science and Engineering. (Wiley, 2011).

[b9] Würfel P., Finkbeiner S. & Daub E. Generalized Planck’s radiation law for luminescence via indirect transitions. Appl. Phys. A 60, 67–70 (1995).

[b10] Putnam M. C. *et al.* Si microwire-array solar cells. Energy Environ. Sci. 3, 1037–1041 (2010).

[b11] Cheng Y. *et al.* Self-Assembled Wire Arrays and ITO Contacts for Silicon Nanowire Solar Cell Applications. Chin. Phys. Lett. 28, 035202 (2011).

[b12] Wang J., Li Z., Singh N. & Lee S. Highly-ordered vertical Si nanowire/nanowall decorated solar cells. Opt. Express 19, 23078; 10.1364/OE.19.023078 (2011). 22109187

[b13] Jung J.-Y., Zhou K., Bang J. H. & Lee J.-H. Improved Photovoltaic Performance of Si Nanowire Solar Cells Integrated with ZnSe Quantum Dots. J. Phys. Chem. C 116, 12409–12414 (2012).

[b14] Huang B.-R., Yang Y.-K., Lin T.-C. & Yang W.-L. A simple and low-cost technique for silicon nanowire arrays based solar cells. Sol. Energy Mater. Sol. Cells 98, 357–362; 10.1016/j.solmat.2011.11.031 (2012).

[b15] Kendrick C. E. *et al.* Radial junction silicon wire array solar cells fabricated by gold-catalyzed vapor-liquid-solid growth. Appl. Phys. Lett. 97, 143108 (2010).

[b16] Nguyen H. P. T., Chang Y.-L., Shih I. & Mi Z. InN p-i-n Nanowire Solar Cells on Si. IEEE J. Sel. Top. Quantum Electron. 17, 1062–1069; 10.1109/JSTQE.2010.2082505 (2011).

[b17] Mariani G., Scofield A. C., Hung C.-H. & Huffaker D. L. GaAs nanopillar-array solar cells employing *in situ* surface passivation. Nat. Commun. 4, 1497; 10.1038/ncomms2509 (2013). 23422665 PMC3586731

[b18] Cirlin G. E. *et al.* Photovoltaic Properties of p-Doped GaAs Nanowire Arrays Grown on n-Type GaAs(111)B Substrate. Nanoscale Res. Lett. 5, 360–363; 10.1007/s11671-009-9488-2 (2009). 20672038 PMC2894346

[b19] Nakai E., Yoshimura M., Tomioka K. & Fukui T. GaAs/InGaP Core-Multishell Nanowire-Array-Based Solar Cells. Jpn. J. Appl. Phys. 52, 055002 (2013).

[b20] Mariani G. *et al.* Patterned Radial GaAs Nanopillar Solar Cells. Nano Lett. 11, 2490–2494; 10.1021/nl200965j (2011). 21604750

[b21] Wallentin J. *et al.* InP Nanowire Array Solar Cells Achieving 13.8% Efficiency by Exceeding the Ray Optics Limit. Science 339, 1057–1060 (2013). 23328392 10.1126/science.1230969

[b22] Cui Y. *et al.* Efficiency Enhancement of InP Nanowire Solar Cells by Surface Cleaning. Nano Lett. 13, 4113–4117; 10.1021/nl4016182 (2013). 23898896

[b23] Yoshimura M., Nakai E., Tomioka K. & Fukui T. Indium Phosphide Core-Shell Nanowire Array Solar Cells with Lattice-Mismatched Window Layer. Appl. Phys. Express 6, 052301 (2013).

[b24] Goto H. *et al.* Growth of Core-Shell InP Nanowires for Photovoltaic Application by Selective-Area Metal Organic Vapor Phase Epitaxy. Appl. Phys. Express 2, 035004 (2009).

[b25] Hirst L. C. & Ekins-Daukes N. J. Fundamental losses in solar cells. Prog. Photovolt. Res. Appl. 19, 286–293; 10.1002/pip.1024 (2011).

[b26] Polman A. & Atwater H. A. Photonic design principles for ultrahigh-efficiency photovoltaics. Nat. Mater. 11, 174–177 (2012). 22349847 10.1038/nmat3263

[b27] Rau U., Paetzold U. W. & Kirchartz T. Thermodynamics of light management in photovoltaic devices. Phys. Rev. B 90, 035211 (2014).

[b28] Henry C. Limiting efficiencies of ideal single and multiple energy gap terrestrial solar cells. J. Appl. Phys. 51, 4494 (1980).

[b29] Ruppel W. & Wurfel P. Upper limit for the conversion of solar energy. *Electron Devices IEEE Trans*. On 27, 877–882; 10.1109/T-ED.1980.19950 (1980).

[b30] Markvart T. Thermodynamics of losses in photovoltaic conversion. Appl. Phys. Lett. 91, 064102 (2007).

[b31] Green M. A. Limits on the open-circuit voltage and efficiency of silicon solar cells imposed by intrinsic Auger processes. Electron Devices IEEE Trans. On 31, 671–678; 10.1109/T-ED.1984.21588 (1984).

[b32] Campbell P. & Green M. A. The limiting efficiency of silicon solar cells under concentrated sunlight. Electron Devices IEEE Trans. On 33, 234–239; 10.1109/T-ED.1986.22472 (1986).

[b33] Araujo G. L. & Martí A. Absolute limiting efficiencies for photovoltaic energy conversion. Sol. Energy Mater. Sol. Cells 33, 213–240; 10.1016/0927-0248(94)90209-7 (1994).

[b34] Braun A., Katz E. A., Feuermann D., Kayes B. M. & Gordon J. M. Photovoltaic performance enhancement by external recycling of photon emission. Energy Environ. Sci. 6, 1499 (2013).

[b35] Kosten E. D., Kayes B. M. & Atwater H. A. Experimental demonstration of enhanced photon recycling in angle-restricted GaAs solar cells. Energy Environ. Sci. 7, 1907 (2014).

[b36] Araújo G. L. & Martí A. Electroluminescence coupling in multiple quantum well diodes and solar cells. Appl. Phys. Lett. 66, 894–895 (1995).

[b37] Liu V. & Fan S. S4: A free electromagnetic solver for layered periodic structures. Comput. Phys. Commun. 183, 2233–2244; 10.1016/j.cpc.2012.04.026 (2012).

[b38] Sandhu S., Yu Z. & Fan S. Detailed balance analysis of nanophotonic solar cells. Opt. Express 21, 1209; 10.1364/OE.21.001209 (2013). 23389013

[b39] Sandhu S., Yu Z. & Fan S. Detailed Balance Analysis and Enhancement of Open-Circuit Voltage in Single-Nanowire Solar Cells. Nano Lett. 14, 1011–1015; 10.1021/nl404501w (2014). 24479660

[b40] Anttu N. Shockley-Queisser Detailed Balance Efficiency Limit for Nanowire Solar Cells. ACS Photonics 2, 446–453; 10.1021/ph5004835 (2015).

[b41] Green M. A., Emery K., Hishikawa Y., Warta W. & Dunlop E. D. Solar cell efficiency tables (version 43). Prog. Photovolt. Res. Appl. 22, 1–9; 10.1002/pip.2452 (2014).

[b42] Kayes B. M., Atwater H. A. & Lewis N. S. Comparison of the device physics principles of planar and radial p-n junction nanorod solar cells. J. Appl. Phys. 97, 114302 (2005).

[b43] Colombo C., Heibeta M., Gratzel M. & Morral A. F. i. Gallium arsenide p-i-n radial structures for photovoltaic applications. Appl. Phys. Lett. 94, 173108 (2009).

[b44] Kelzenberg M. D. *et al.* Enhanced absorption and carrier collection in Si wire arrays for photovoltaic applications. Nat. Mater. 9, 239–244 (2010). 20154692 10.1038/nmat2635

[b45] Schnitzer I., Yablonovitch E., Caneau C. & Gmitter T. J. Ultrahigh spontaneous emission quantum efficiency, 99.7% internally and 72% externally, from AlGaAs/GaAs/AlGaAs double heterostructures. Appl. Phys. Lett. 62, 131–133 (1993).

[b46] Miller O. D., Yablonovitch E. & Kurtz S. R. Strong Internal and External Luminescence as Solar Cells Approach the Shockley—Queisser Limit. IEEE J. Photovolt. 2, 303–311 (2012).

[b47] Joyce H. J. *et al.* Ultralow Surface Recombination Velocity in InP Nanowires Probed by Terahertz Spectroscopy. Nano Lett. 12, 5325–5330; 10.1021/nl3026828 (2012). 22962963

[b48] Joyce H. J. *et al.* Electronic properties of GaAs, InAs and InP nanowires studied by terahertz spectroscopy. Nanotechnology 24, 214006 (2013). 23619012 10.1088/0957-4484/24/21/214006

[b49] Shen Y. *et al.* Optical Broadband Angular Selectivity. Science 343, 1499–1501 (2014). 24675959 10.1126/science.1249799

[b50] Shen Y. *et al.* Metamaterial broadband angular selectivity. Phys. Rev. B 90, 125422 (2014).

